# Identification of Ferroptosis-Related Genes in Schizophrenia Based on Bioinformatic Analysis

**DOI:** 10.3390/genes13112168

**Published:** 2022-11-20

**Authors:** Shunkang Feng, Jun Chen, Chunhui Qu, Lu Yang, Xiaohui Wu, Shuo Wang, Tao Yang, Hongmei Liu, Yiru Fang, Ping Sun

**Affiliations:** 1 Qingdao University Medical College, Qingdao 266071, China; 2 Qingdao Mental Health Center, Qingdao 266034, China; 3 Clinical Research Center and Division of Mood Disorders, Shanghai Mental Health Center, Shanghai Jiao Tong University School of Medicine, Shanghai 200030, China; 4 CAS Center for Excellence in Brain Science and Intelligence Technology, Shanghai 200031, China; 5 Shanghai Key Laboratory of Psychotic Disorders, Shanghai 201108, China

**Keywords:** ferroptosis, schizophrenia, GEO, bioinformatic

## Abstract

The purpose of this study is to explore the correlation between ferroptosis-related genes and schizophrenia in order to explore the new direction of diagnosis and treatment of schizophrenia. We screened the datasets related to schizophrenia from the Gene Expression Comprehensive Database (GEO) and obtained ferroptosis-related genes from the FerrDB database. Bioinformatics methods were used to analyze differentially expressed genes (DEGs) and genes associated with ferroptosis-related between schizophrenia patients and healthy controls. On this basis, the hub genes were finally screened by enrichment analysis and PPI interaction analysis. Hub genes associated with ferroptosis were validated using other schizophrenia datasets in the GEO database. Finally, the hub gene-microRNA (miRNA), gene-transcription factor interaction network was constructed, and three ferroptosis-related hub genes (TP53, VEGFA and PTGS2) were screened. The validation results of these three genes in other datasets also support this conclusion. A miRNA: hsa-mir-16-5p was found to be related to the three hub genes, and pPHF8, SAP30 and lKDM5B were identified as common regulators of the three hub genes. Our results indicate that TP53, VEGFA and PTGS2 are significantly associated with schizophrenia, and may be ferroptosis-related markers of the disease.

## 1. Introduction

Schizophrenia is a common mental disease characterized by progressive cognitive impairment in attention, working memory and executive function. Although no clear etiology of schizophrenia has been found, previous studies have confirmed that the disease has strong genetic and familial transmission, and its pathogenesis is highly related to genes [1].

Ferroptosis is a regulatory cell death, and the immoderate accumulation of iron structured lipid reactive oxygen species (ROS) and consumption of polyunsaturated fatty acids are its foremost mechanisms [2]. Previous studies have shown that there is a significant correlation between the ferroptosis process and a variety of neuropsychiatric diseases [3], so ferroptosis-related genes may also participate in the pathogenesis of schizophrenia, but there is no research on the correlation between ferroptosis-related genes and schizophrenia. The motive of this study is to discover the relationship between ferroptosis-related genes and schizophrenia through the existing data in the GEO database and FerrDB database.

## 2. Research Data and Bioinformatics Methods

### 2.1. Screening of Research Data

The data of this study are mainly from existing schizophrenia datasets in the GEO database (https://www.ncbi.nlm.nih.gov/geo/, accessed on 15 November 2022) [4]. The two GEO datasets included in the study were all microarray arrays based on the GPL570[HG-U133_Plus_2] Affymetrix human genome U133 Plus 2.0 array. GSE27383 was derived from peripheral blood mononuclear cells of 43 patients with schizophrenia and 29 healthy individuals. Samples of GSE21138 were taken from the prefrontal cortex of 29 schizophrenia patients and 30 healthy people. Ferroptosis-related genes are from the ferroptosis-related database (FerrDB) (http://www.zhounan.org/ferrdb/index.html, accessed on 15 November 2022) [5], including driver, suppressor and marker. GSE27383 was used to obtain common DEGs by crossing with genes in FerrDB, and GSE21138 was used for further screening and validation of hub genes.

### 2.2. Identification of DEGs Related to Ferroptosis

Differential expression analysis of the two gene expression profiles was performed using the GEO2R online analysis tool. Compared with other diseases, mental diseases are more special. At present, there is no specific single gene that can cause schizophrenia. In order to avoid omitting more important DEGs, *p* < 0.05 and |log FC| > 0.1 are used as the screening criterion for DEGs. DEGs with log FC < 0 were thought to be down-regulated genes, whereas DEGs with log FC > 0 were thought to be up-regulated genes. The data of two gene expression profiles (GSE27383 and GSE21138) were screened for DEGs, and then volcano mapping tools were used (https://www.xiantao.love/products, accessed on 15 November 2022) to draw the volcano map of GSE27383. Subsequently, using the Venn map network tool (http://bioinformatics.psb.ugent.be/webtools/Venn/, accessed on 15 November 2022), the overlap between the DEGs of GSE27383 and 259 ferroptosis-related genes was examined. They are ferroptosis-related genes that may participate in the pathogenesis of schizophrenia.

### 2.3. GO and KEGG Enrichment Analysis

The selected DEGs and hub genes were analyzed for GO and KEGG enrichment analysis using David (6.8) (https://david.ncifcrf, accessed on 15 November 2022). GO has three levels of analysis: molecular function (MF), cellular component (CC), and biological process (BP). A widespread database used to investigate illnesses, chemicals, medications, biological processes, and genomes is called KEGG. When DEG met *p* < 0.05 and count ≥ 10 in the above two analyses, it had statistical significance in this study. Weshengxin (http://www.bioinformatics.com.cn, accessed on 15 November 2022), a free online application for data processing and visualization, was used to create the bubble diagram.

### 2.4. PPI Network Construction and Hub Gene Screening

Build a protein-protein interaction (PPI) network using the free, open-source STRING database (https://string-db.org/, accessed on 15 November 2022). To assess PPI, import the filtered DEGs into the STRING database. Create a visual network of PPIs using the Cytoscape program (https://cyto scape.org, accessed on 15 November 2022), then use Cytohubba to scan hub genes.

### 2.5. Validation of Hub Genes

The GEO dataset GSE21138 was used to validate the hub genes. The expression of hub genes in brain tissue was examined using the statistical program SPSS 26.0. Unpaired *t* test was the statistical approach employed, and *p*-values of 0.05 or below were utilized to determine statistical significance. The results were visualized with the help of violin charts using an online chart drawing tool (https://www.xiantao.love/products, accessed on 15 November 2022). The diagnostic utility of hub genes in schizophrenia was then assessed using an online receiver operating characteristic (ROC) curve design tool (https://www.xiantao.love/products, accessed on 15 November 2022).

### 2.6. Construction of miRNA (or TF)–Hub Gene Network

Networkanalyst (https://www.networkanalyst.ca/, accessed on 15 November 2022) created interaction networks between genes-miRNAs and genes-transcription factors [6]. The parameters used were as follows: specify organism: H. sapiens (human); set ID type: Official Gene Symbol; Gene-miRNA interaction database: miRTarBasev8.0, TarBase v8.0 and miRecords databases; and Gene-TF interaction database: ENCODE, JASPAR, and ChEA databases.

## 3. Results

### 3.1. Ferroptosis-Related DEGs in Schizophrenia

**Figure 1:**
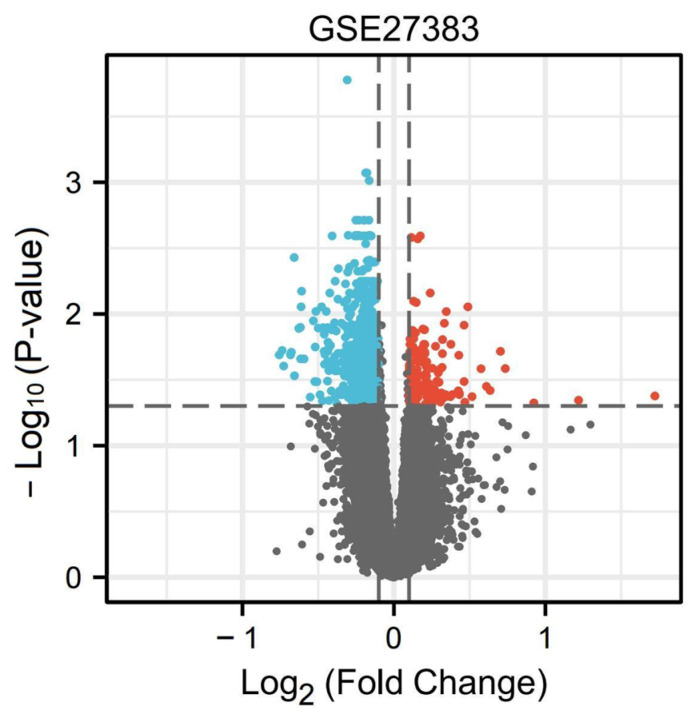
Identification of DEGs between schizophrenia patients and healthy people.

In the GEO database, we screened GSE27383 from schizophrenia related datasets for intersection with ferroptosis-related genes. Through the online tool GEO2R, GSE27383 was analyzed according to the standard of *p* < 0.05 and |log FC| > 0.1, and a total of 3338 DEGs were obtained. A volcano map was used to display the DEGs of samples with schizophrenia and healthy samples. All nodes on the volcano map indicate the DEGs between the healthy and schizophrenia samples. The nodes that fulfill the screening requirements (*p* < 0.05 and |log FC| > 0.1) differ considerably. Red represents up-regulation of this gene expression, while blue represents down-regulation. (Figure 1).

**Figure 2:**
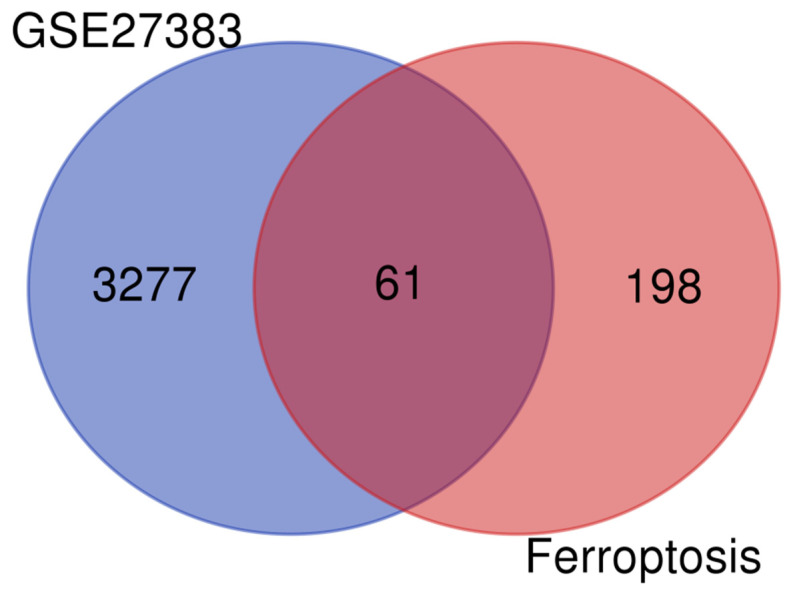
Venn diagram of DEGs from GSE27383 and ferroptosis-related genes.

Following that, a Venn diagram was used to determine the intersection findings between the aforementioned DEGs and 259 genes relevant to ferroptosis. The overlap of these two sets of data has 61 similar DEGs, as seen in Figure 2. The classification of these 61 common DEGs in ferroptosis is shown in Table 1.

### 3.2. GO Analysis and KEGG Pathway Analysis

**Figure 3:**
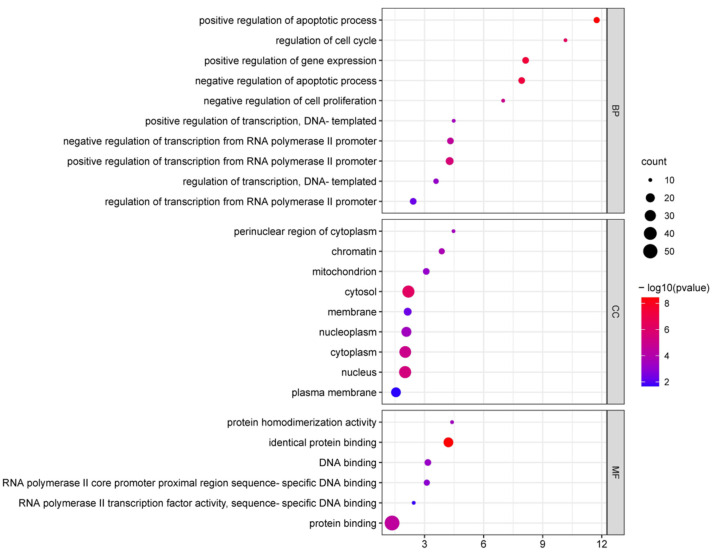
Significant GO terms of ferroptosis-related DEGs.

Following the examination of common DEGs, the analysis findings were scrutinized using the *p* < 0.05 and count ≥ 10 criterion. GO enrichment analysis was performed on the 61 DEGs. The procedure has three levels: molecular function (MF), cell component (CC), and biological process (BP). The outcomes are displayed in Figure 3.

In BP, ferroptosis-related DEGs were enriched in positive regulation of the apoptotic process. This included positive regulation of gene expression; negative regulation of apoptotic processes; regulation of the cell cycle; positive regulation of transcription from RNA polymerase II promoter; negative regulation of cell proliferation; negative regulation of transcription from RNA polymerase II promoter; positive regulation of transcription, DNA-templated; regulation of transcription, DNA-templated; regulation of transcription from RNA polymerase II promoter.

In terms of the CC, they were mainly enriched in the cytosol, nucleus, cytoplasm, chromatin, perinuclear region of cytoplasm, nucleoplasm, mitochondrion, membrane, plasma membrane.

For MF, these genes were enriched in identical protein binding: protein binding; protein homodimerization activity; DNA binding; RNA polymerase II core promoter proximal region sequence-specific DNA binding; RNA polymerase II transcription factor activity, sequence-specific DNA binding.

**Figure 4:**
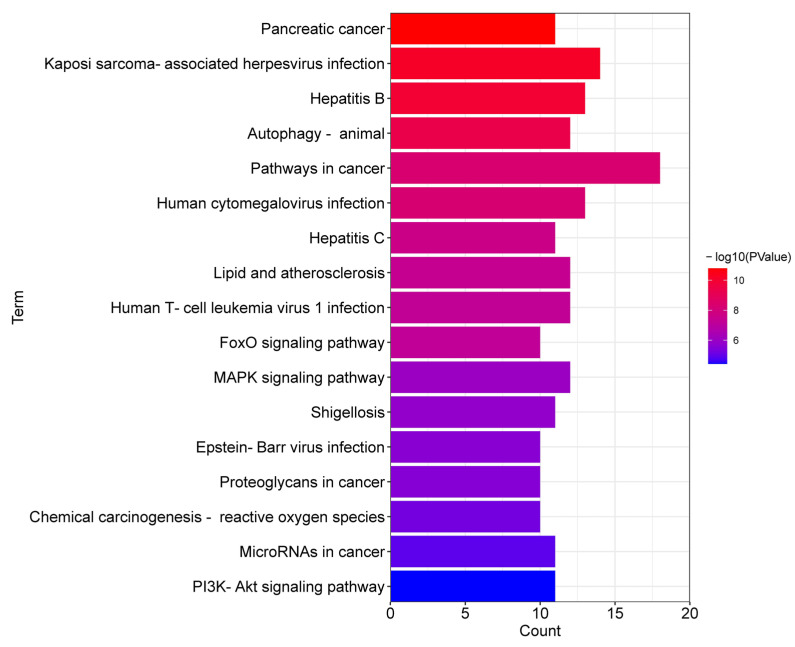
Significant KEGG pathways of ferroptosis-related DEGs.

In addition, the results of KEGG pathway analysis of ferroptosis-related DEGs are shown in Figure 4. They were mainly enriched in pancreatic cancer; Kaposi sarcoma-associated herpesvirus infection; hepatitis B; autophagy—animal; pathways in cancer; human cytomegalovirus infection; hepatitis C; lipid and atherosclerosis; Human T-cell leukemia virus 1 infection; FoxO signaling pathway; MAPK signaling pathway; Shigellosis; Epstein-Barr virus infection; proteoglycans in cancer; chemical carcinogenesis—reactive oxygen species; microRNAs in cancer; PI3K-Akt signaling pathway.

### 3.3. PPI Network Design and Hub Gene Identification

**Figure 5:**
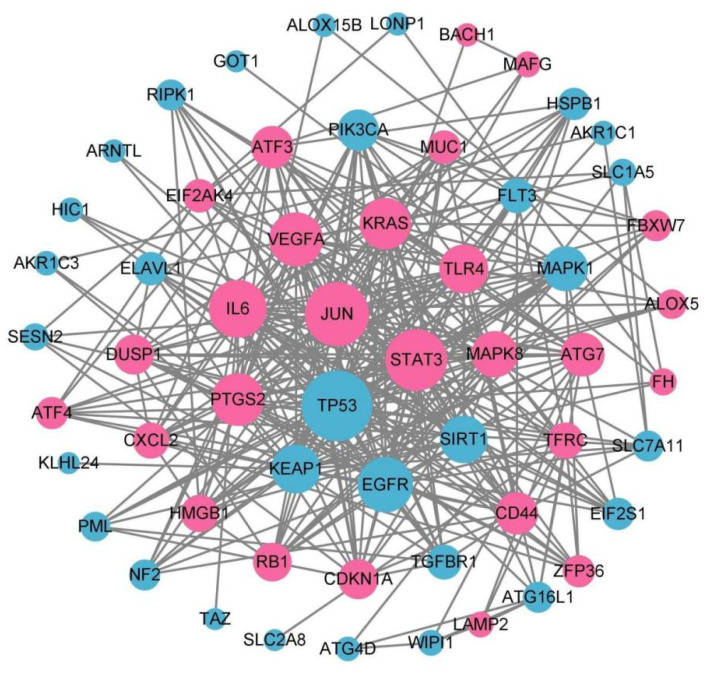
PPI network constructed with ferroptosis-related DEGs. Red indicates genes that are up-regulated, whereas blue indicates genes that are down-regulated.

STRING database was used to retrieve 61 genes. The PPI network information includes 61 nodes and 341 edges. The network diagram was constructed with Cytoscape software (3.9.0) (Figure 5).

**Figure 6:**
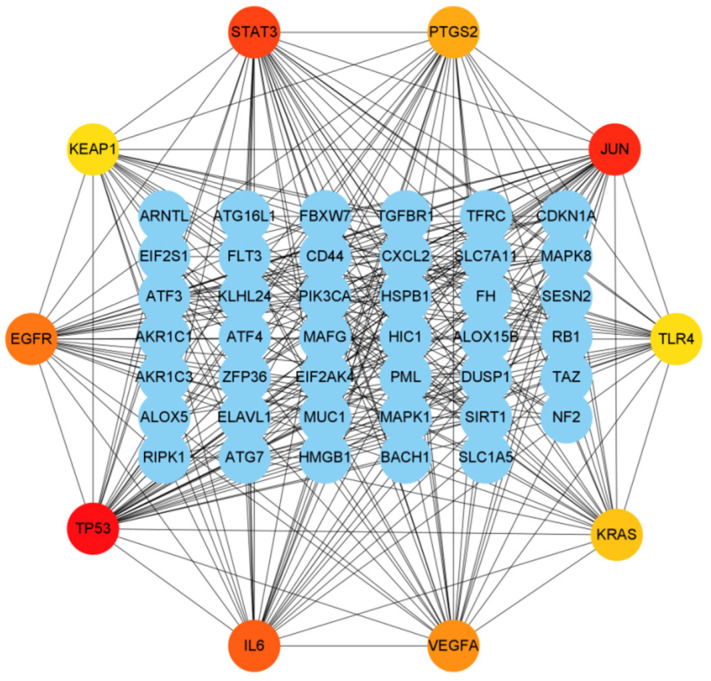
PPI network created using additional DEGs and 10 hub genes. The weight of a hub gene across the network increases with the hue of the gene.

After that, Cytohubba was used to continue the screening procedure for hub genes. According to the degree ranking, the top 10 genes were chosen, as shown in Figure 6.

### 3.4. Validation of Hub Genes

**Figure 7:**
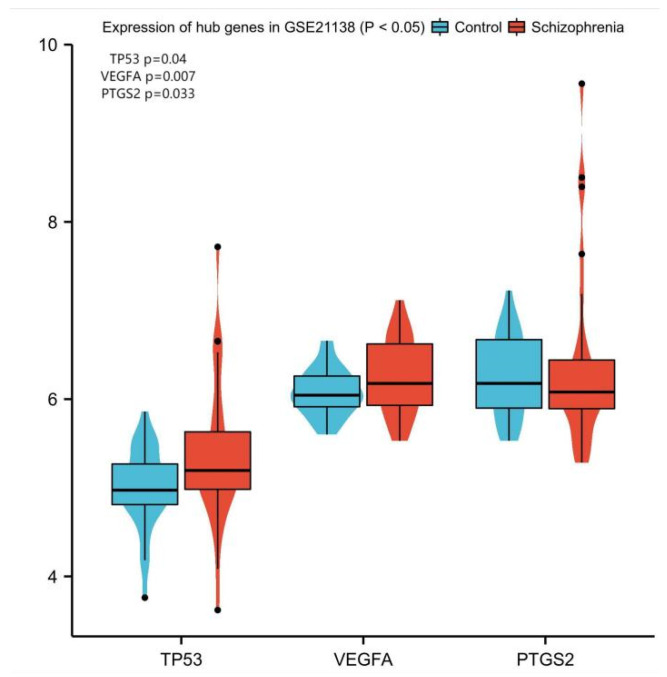
Expression of TP53, VEGFA and PTGS2 in brain tissue (*p* < 0.05).

To confirm hub gene expression in brain tissue, GSE21383 was utilized. According to the findings, only three of the ten hub genes—TP53, VEGFA, and PTGS2—had substantially different expression levels in brain tissue between schizophrenia patients and healthy controls (*p* < 0.05, Figure 7). Among them, VEGFA was up-regulated in both GSE27383 and GSE21138.

**Figure 8:**
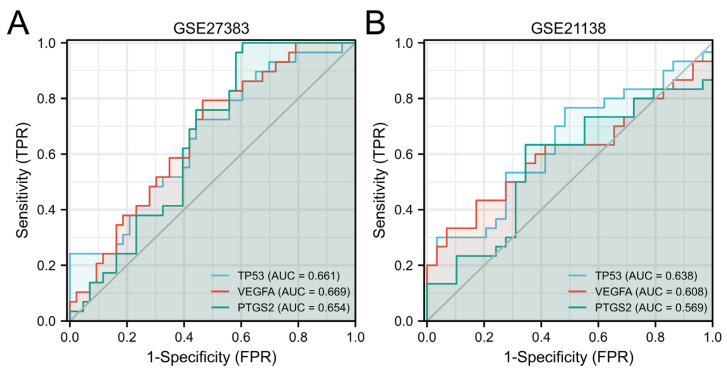
A diagnostic assessment of three hub genes. (**A**). GSE27383; (**B**). GSE21138.

**Figure 9:**
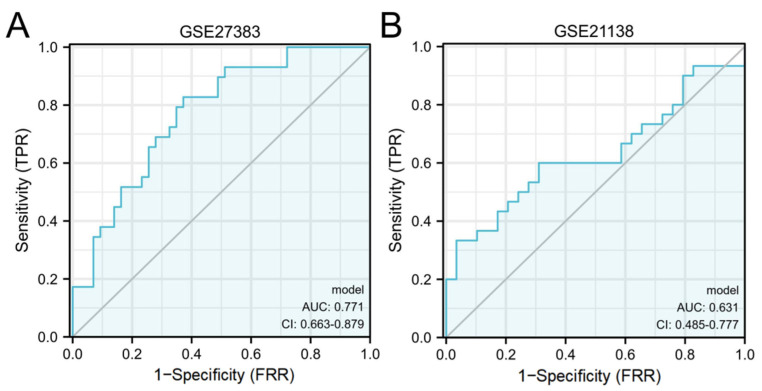
Examination of joint markers for diagnosis. (**A**). GSE27383; (**B**). GSE21138.

After that, the diagnostic value of the above three hub genes was verified by using the ROC curve. As separate diagnostic indicators, the results are shown in Figure 8. When the three hub genes are used as joint indicators, the outcomes are displayed in Figure 9.

When three hub genes were utilized as distinct diagnostic indications, the ROC curve data demonstrated that TP53 measured in the GSE27383 was 0.661 (95% confidence interval (CI), 0.533–0.789), VEGFA was 0.669 (95% confidence interval (CI), 0.544–0.794) and PTGS2 was 0.654 (95% confidence interval (CI), 0.529–0.780). In GSE21138, TP53 was 0.638 (95% confidence interval (CI), 0.494–0.782), VEGFA was 0.608 (95% confidence interval (CI), 0.460–0.756) and PTGS2 was 0.569 (95% confidence interval (CI), 0.417–0.720).

When 3 genes were used as a joint indicator, the diagnostic value in GSE27383 was 0.771 (95% confidence interval (CI), 0.663–0.879) and the diagnostic value in GSE27383 was 0.631 (95% confidence interval (CI), 0.485–0.777).

### 3.5. Results of Gene-miRNA and Gene-TF

**Figure 10:**
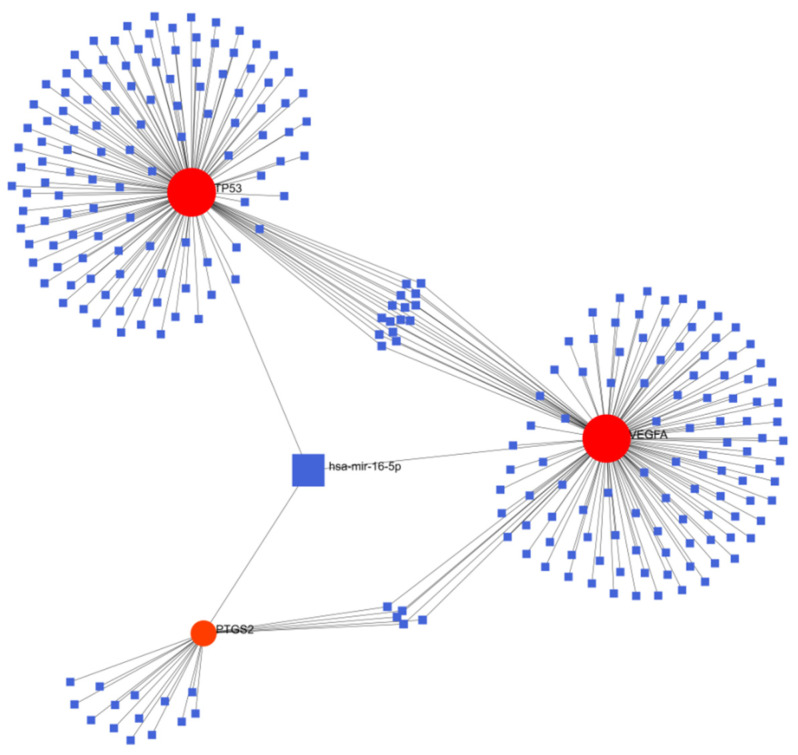
Interactions among 3 hub genes and miRNAs.

Gene and miRNA or TF interaction networks were generated by Network analysis. The gene-miRNA network of TP53, VEGFA and PTGS2 was constructed, as shown in Figure 10. There were 257 nodes and 276 edges, among which hsa-mir-16-5p was related to the three hub genes. This indicates that hsa-mir-16-5p can simultaneously regulate the expression of TP53, VEGFA and PTGS2.

**Figure 11:**
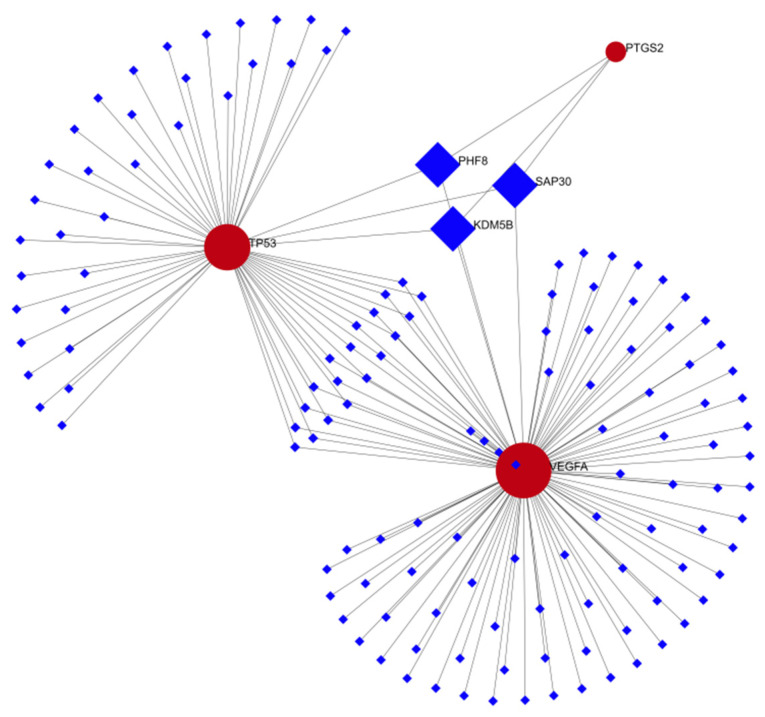
Interactions among 3 hub genes and transcription factors.

After this, the TP53, VEGFA, and PTGS2 hub genes’ gene-TF network was built, and PHD finger protein 8 (PHF8), Sin3a associated protein 30 (SAP30) and Iysine demethylase 5B (KDM5B) were identified as the common regulators of the three hub genes (Figure 11).

## 4. Discussion

Previous research results suggest that the ferroptosis is significantly related to a variety of neuropsychiatric diseases. Schizophrenia, as a common psychiatric disease, has a greater relationship between its pathogenesis and genes. The pathophysiology of schizophrenia may possibly include the ferroptosis. In this study, bioinformatics analysis, GEO database and FerrDB were used to explore ferroptosis-related genes that play a role in the pathogenesis of schizophrenia. A total of 61 ferroptosis-related genes related to schizophrenia were preliminarily screened. According to the classification of 61 DEGs, there are 25 marker genes, 21 driver genes and 15 suppressor genes. Most of the ferroptosis-related genes in schizophrenia belong to the marker and driver categories, while the suppressors are relatively few. The results of enrichment analysis indicated that they were mainly enriched in the pathways related to cell cycle, transcription and protein construction. The results suggest that ferroptosis-related genes may affect the disease progression of schizophrenia through the above processes.

Through subsequent screening, three prominent hub genes were finally identified: TP53, VEGFA and PTGS2. The full name of TP53 is tumor protein p53, which encodes a tumor suppressor protein. The encoded protein is mainly involved in the regulation of cell stress response and target gene expression, and ultimately induces cell cycle arrest, apoptosis, aging, DNA repair or metabolic changes [7]. TP53 is located on chromosome 17p13.1, which was previously reported to be significantly associated with schizophrenia [8]. In a previous study including 701 patients and 695 controls, the results also suggested that TP53 could increase the susceptibility to schizophrenia [9]. In another study on the polymorphism of this gene, the results also indicated that there was a significant association between TP53 and schizophrenia. In this study, the researchers proposed that the mechanism of this gene leading to the pathogenesis of schizophrenia might be related to neurodevelopment and apoptosis [10]. These research findings concur with those from this study, which suggest that TP53 may have a role in the pathophysiology of schizophrenia, and the enrichment analysis also suggests that TP53 may participate in the process of apoptosis.

The full name of VEGFA is Vascular endothelial growth factor A, which is a member of the PDGF/VEGF growth factor family. Vascular endothelial cells are stimulated to proliferate and migrate by this growth factor [7]. A study published in 2021 claimed that VEGFA, as an angiogenic and neurotrophic factor, can participate in the regulation of cerebral blood volume and flow in patients with schizophrenia. This leads to the changes of cognitive ability and brain function in schizophrenic patients [11]. Many earlier studies are consistent with the results of this study, and all support that VEGFA can affect the cognitive function of patients [12,13]. The above results also support the results of this study: VEGFA is significantly related to schizophrenia.

Also known as prostaglandin-endoperoxide synthase 2, PTGS2 has this complete name. The primary enzyme in prostaglandin manufacture, PTGS, commonly known as cyclooxygenase, functions as both a peroxidase and a dioxygenase [7]. This gene is related to the inflammatory response to some extent, and the pathogenesis of schizophrenia is also related to the inflammatory response. This gene may indirectly affect the development of schizophrenia by regulating the inflammatory response. A study published in 2004 mentioned that PTGS2 can affect the susceptibility to schizophrenia [14]. The results also support the conclusions of this study.

ROC curve results showed that when the three hub genes were used as independent diagnostic indicators, they were all of diagnostic value for schizophrenia. As a joint indicator, the diagnostic value was slightly improved. Although the two results are not of very significant diagnostic value, they still have important significance for polygenic diseases such as schizophrenia. In a previous study, researchers explored early diagnostic indicators of bipolar disorder through oxidative stress injury biomarker model. This study also provides a new idea for research into computer algorithm integration, and is expected to provide a new idea for the early diagnosis of bipolar disorder [15].

The above hub genes are all ferroptosis-related genes, and the results of this study suggest that the three hub genes have certain effects on the pathogenesis of schizophrenia; previous studies also support this conclusion. Enrichment analysis showed that the hub genes were mainly enriched in the cell cycle and apoptosis pathway, which overlapped with the process of ferroptosis. Therefore, the process of ferroptosis may play an important role in the pathogenesis of schizophrenia.

The subsequent construction of a gene-miRNA network showed a node associated with the three hub genes. Hsa-mir-16-5p can regulate the three genes at the same time. Therefore, further research on this miRNA may be of great significance to explore the pathogenesis and treatment of schizophrenia. PHF8, SAP30 and KDM5B as co regulators of the three hub genes may provide a new idea for the treatment of schizophrenia.

However, there are still shortcomings in this study. Although there are differences in the expression of TP53 and PTGS2 between schizophrenia patients and the control group, the expression levels in the two datasets are not consistent. TP53 was lower in GSE27383 than in the control group, but higher in GSE21138. PTGS2 was highly expressed in GSE27383 compared with the healthy group, but was poorly expressed in GSE21138. This difference may be caused by the different sample sources of the two datasets. The expression data of GSE27383 is from peripheral blood mononuclear cells and GSE21138 is from brain tissue.

Nevertheless, the results are consistent, suggesting that the three ferroptosis-related genes are indeed significantly associated with schizophrenia. Further understanding the relationship between ferroptosis-related genes and schizophrenia will help to explore its etiology and provide new ideas for future diagnosis and treatment.

## 5. Conclusions

The results of this study suggest that the three ferroptosis-related genes, namely, TP53, VEGFA and PTGS2 are significantly related to schizophrenia, and that ferroptosis may be involved in the pathogenesis of the disease.

## Figures and Tables

**Table 1:** Classification of these 61 common DEGs.

Marker	Driver	Suppressor
CXCL2	FBXW7	JUN
PTGS2	TLR4	CD44
KRAS	BACH1	ZFP36
TFRC	MAPK8	CDKN1A
ALOX5	ATG7	FH
IL6	PIK3CA	LAMP2
VEGFA	GOT1	RB1
ATF3	TAZ	STAT3
EIF2AK4	SIRT1	MUC1
DUSP1	TGFBR1	NF2
HMGB1	LONP1	PML
MAFG	TP53	ARNTL
ATF4	SLC1A5	AKR1C1
MAPK1	ATG16L1	CISD1
RIPK1	DNAJB6	AKR1C3
ELAVL1	ATG4D	
SLC2A8	KEAP1	
SLC7A11	WIPI1	
AGPAT3	EGFR	
HIC1	ALOX15B	
HSPB1	FLT3	
KLHL24		
SESN2		
ZNF419		
EIF2S1		

## Data Availability

All data are obtained from public data in the GEO database.

## References

[1] van de Leemput J., Hess J.L., Glatt S.J., Tsuang M.T. (2016). Genetics of Schizophrenia: Historical Insights and Prevailing Evidence. Adv. Genet..

[2] Dixon S.J., Lemberg K.M., Lamprecht M.R., Skouta R., Zaitsev E.M., Gleason C.E., Patel D.N., Bauer A.J., Cantley A.M., Yang W.S. (2012). Ferroptosis: An iron-dependent form of nonapoptotic cell death. Cell.

[3] Weiland A., Wang Y., Wu W., Lan X., Han X., Li Q., Wang J. (2019). Ferroptosis and Its Role in Diverse Brain Diseases. Mol. Neurobiol..

[4] Edgar R., Domrachev M., Lash A.E. (2002). Gene Expression Omnibus: NCBI gene expression and hybridization array data repository. Nucleic Acids Res..

[5] Zhou N., Bao J. (2020). FerrDb: A manually curated resource for regulators and markers of ferroptosis and ferroptosis-disease associations. Database J. Biol. Databases Curation.

[6] Zhou G., Soufan O., Ewald J., Hancock R.E.W., Basu N., Xia J. (2019). NetworkAnalyst 3.0: A visual analytics platform for comprehensive gene expression profiling and meta-analysis. Nucleic Acids Res..

[7] Fagerberg L., Hallström B.M., Oksvold P., Kampf C., Djureinovic D., Odeberg J., Habuka M., Tahmasebpoor S., Danielsson A., Edlund K. (2014). Analysis of the human tissue-specific expression by genome-wide integration of transcriptomics and antibody-based proteomics. Mol. Cell. Proteom. MCP.

[8] Freedman R., Leonard S., Olincy A., Kaufmann C.A., Malaspina D., Cloninger C.R., Svrakic D., Faraone S.V., Tsuang M.T. (2001). Evidence for the multigenic inheritance of schizophrenia. Am. J. Med. Genet..

[9] Yang Y., Xiao Z., Chen W., Sang H., Guan Y., Peng Y., Zhang D., Gu Z., Qian M., He G. (2004). Tumor suppressor gene TP53 is genetically associated with schizophrenia in the Chinese population. Neurosci. Lett..

[10] Ni X., Trakalo J., Valente J., Azevedo M.H., Pato M.T., Pato C.N., Kennedy J.L. (2005). Human p53 tumor suppressor gene (TP53) and schizophrenia: Case-control and family studies. Neurosci. Lett..

[11] Rampino A., Annese T., Torretta S., Tamma R., Falcone R.M., Ribatti D. (2021). Involvement of vascular endothelial growth factor in schizophrenia. Neurosci. Lett..

[12] Jung J., Kim S., Yoon K., Moon Y., Roh D., Lee S., Choi K., Jung J., Kim D. (2015). The effect of depression on serum VEGF level in Alzheimer’s disease. Dis. Mrk..

[13] Newton S.S., Fournier N.M., Duman R.S. (2013). Vascular growth factors in neuropsychiatry. Cell. Mol. Life Sci. CMLS.

[14] Wei J., Hemmings G.P. (2004). A study of a genetic association between the PTGS2/PLA2G4A locus and schizophrenia. Prostaglandins Leukot. Essent. Fat. Acids.

[15] Niu Z., Wu X., Zhu Y., Yang L., Shi Y., Wang Y., Qiu H., Gu W., Wu Y., Long X. (2022). Early Diagnosis of Bipolar Disorder Coming Soon: Application of an Oxidative Stress Injury Biomarker (BIOS) Model. Neurosci. Bull..

